# Interpretation of intrafraction motion review data and method for verification

**DOI:** 10.1002/acm2.13379

**Published:** 2021-09-28

**Authors:** Gavin Graeper, Ashley Cetnar, Ahmet S. Ayan, Michael Weldon

**Affiliations:** ^1^ Department of Radiation Oncology The Ohio State University Wexner Medical Center Columbus Ohio USA

**Keywords:** dual surrogate, Motion management (intrafraction), x‐ray projection

## Abstract

The current clinical interface for Varian's intrafraction motion review (IMR) is limited, providing only qualitative data for review at the treatment console. This study provides a method of extracting and interpreting data from combined log files for quantitative evaluation. Combined log files acquired during patient treatment and a parsing code was developed to scan the combined log file looking for unique identifiers pertaining to the data of interest. We were able to extract clinically relevant parameters from the log files including date and time, gantry angle, expected marker position, found marker position, pixel size, and detection result. This study details how to compare IMR data to Calypso investigating dual‐surrogates for intrafraction monitoring during treatment for other researchers to build on these methods. Understanding data recorded during treatment within the combined log files can be helpful in quality improvement of patient care by retrospectively reviewing intrafraction motion.

## 1 INTRODUCTION

One of the most challenging aspects of radiation therapy treatment is localizing the target. Orthogonal or cone‐beam computed tomography (CBCT) imaging is common for modern linear accelerator‐based (linac) treatment, though soft tissue evaluation can be difficult to evaluate using these methods. To help with these challenges, it is common to insert fiducial markers such as gold seeds into or adjacent to the target to be used as surrogates for localization. These fiducials can also be used to monitor the target during treatment, though more sophisticated tools are required.

For departments using Varian TrueBeam® linacs with Advanced Imaging, Intrafraction Motion Review (IMR) is a tool for monitoring fiducial positions throughout treatment. Fixed points in space with reference to isocenter, known as markers, are placed in reference to the fiducial location during treatment planning. IMR uses the position of the markers to calculate where the fiducials would be expected when taking kilovoltage (kV) images at associated gantry angles during treatment and an algorithm compares these expected locations to the detected fiducial location in the image. The images are taken orthogonal to the treatment axis, and the frequency of the imaging is determined by the user based on gantry angle, time, or number of delivered monitor units.

**FIGURE 1:**
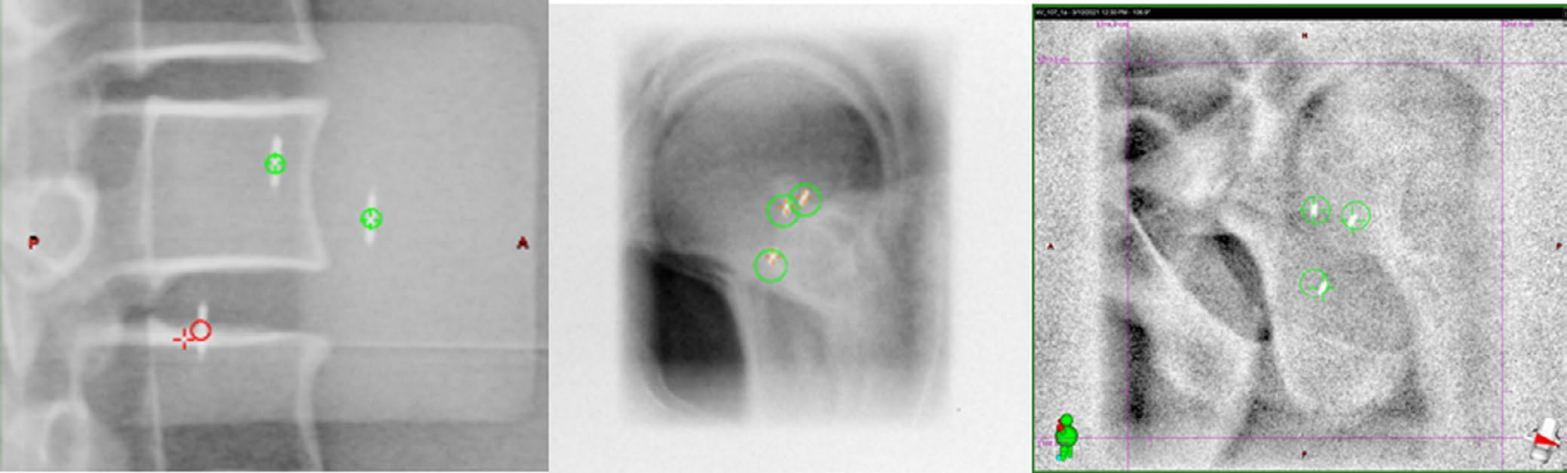
Examples of IMR data displayed for the user at the console. Green denotes within tolerance and red within tolerance. The red detection event on the left shows a misdetection, the crosshair is clearly on the vertebral body instead of the seed

While IMR has been shown to be useful by investigators,^1^, ^2^, ^3^, ^4^, ^5^, ^6^ the current version of IMR (TrueBeam® v2.7 MR3) is not without its limitations. The tolerance information received by the clinical user during treatment is displayed qualitatively as green, red, or orange corresponding to whether the location for each fiducial is detected within the set tolerance, outside of the tolerance, or not found, respectively, as shown in Figure 1. Unfortunately, this information is not currently available for evaluation offline. It can be reviewed real‐time or immediately after treatment at the console but is no longer able to be accessed by the user after closing out the patient's treatment session. The images are available to review offline but do not retain the red/green/orange distinction used for clinical guidance during treatment.

Although it is currently not a user‐friendly method for extraction and analysis, the expected and detected marker location data is recorded in the combined log files. The goal of this work is to share our method for IMR data extraction so that others can improve patient care in their own clinic. We also present our method for comparing IMR data with Calypso data positions for further research.

## 2 METHODS

### 2.1 Data extraction from combined log files

All IMR data is stored in combined log files on the linac console computer, which is compiled daily, archived to last several months, and then periodically deleted. These files are intended for service engineers to access and use, so the data is not exported automatically for clinical operations via PeerSync™ requiring manual extraction from the console computer. Access to the drive where the files are located is limited, so if the user lacks the appropriate rights, it may be necessary to contact the vendor to transfer the files.

**FIGURE 2:**
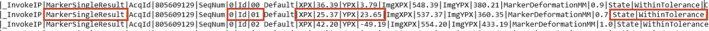
Partial snapshot showing three lines of the log file for found fiducial data. This is specifically for fiducial “00,” X pixel position, Y pixel position, and that it was found to be within the threshold tolerance. “MarkerSingleResult” was used as an identifier specifically meaning that the information on that line of code was for found seeds. Expected seeds used the tag of “ExpectedMarkerPosition.” Gantry rotation and date/time were from “ImageSource” and “ImageAquisition” respectively

**FIGURE 3:**
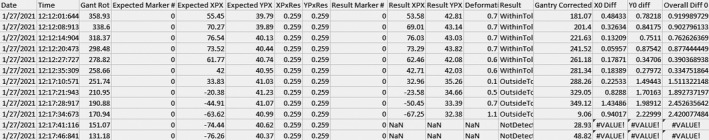
An example of the final format of the data once it has been parsed from the log file. This was saved as a.csv file for easy use in a spreadsheet. The final four columns are not pulled from the log file but are instead calculated using the log file data in the previous columns. Gantry refers to the head of the linac, not the imager, however, its position is stored using the IEC convention so we applied a shift to convert to the Varian coordinates. The final 3 columns show the calculated positional differences in millimeters between expected and found positions for seed 0 for each axis as well as overall

Combined log files track nearly all of the linac's operations throughout the day, making them difficult to extract only the IMR‐relevant data using manual export method as shown in Figure 2. We developed a parsing code that extracts the data of interest and saves it as a comma‐separated text file. Parameters deemed important to acquire from the log files for our study were date/time, gantry angle, expected marker position, found marker position, pixel size, and detection result. An example of relevant log file data compiled into a.csv file is shown in Figure 3. There are many lines pertaining to the IMR imaging sections within the log files, so this work is not meant to be an exhaustive guide for interpretation. If there is other information desired for specific analysis, it is recommended to consult the manufacturer rather than make assumptions about label definitions.

It is recommended that care is taken to ensure the correct location data is being extracted from the log file. For IMR, there are three sets of positions for each fiducial's X and Y coordinates. Labels for maker positions include the expected position noted as “ExpectedMarkerPosition” and found position as “MarkerSingleResult” for data analysis. While “MarkerGeometryCheck” also records fiducial positional data, it is not directly related to found or expected fiducial position and is not needed to determine planned versus treatment absolute fiducial positional differences. If one or more markers are not detected, they are recorded as such (NaN) in the combined log file.

Fiducial position data is stored in terms of pixel location, making it is necessary to obtain pixel size information to convert to the International System of Units. The log file subdivides all positional and pixel information by fiducial and X and Y coordinates. The information collected using this method yields X and Y marker positions relative to the imaging panel. If pixel size is used to convert to units of millimeters, it should be noted that this distance is for the projection at isocenter, not at the imaging panel distance.

Detection result is the classification of the color‐coded results displayed on the console during treatment (within the set tolerance, outside of the tolerance, or not found). The stored data does not record found versus expected fiducial position difference, but the absolute X and Y positions and the detection result (i.e., within or outside tolerance). However, such numerical detection positional differences can be calculated externally.

Combined log files do not store any patient information, so if it is desired to correlate the data to a particular patient, a system for patient identification will need to be developed. To accomplish this, we used a time‐based system such that the time of each beam or arc was recorded during treatment in reference to that particular patient and fraction. Time information is also stored on each line of the log file with millisecond precision. If this is extracted for each segment of image data, it is then straightforward to match time information to patient data.

A step‐by‐step guide of the complete process if performed manually is as follows. Steps 2–7 were automated by the authors for this study to increase the efficiency and accuracy of the data processing. Please note that the unique identifiers may have changed since publication and will vary depending on what data is required for your analysis.

1. Log into the console computer as an admin and copy the combined log files to a drive accessible on your network.
2. Within the log file, locate the first instance of the unique identifier “ImageAcquisition” and copy the date and time from that row.
3. Continuing down the file, the next unique identifier of interest is InvokeIP|ImageSource|AcqId” which represents gantry angle information in this row.
4. Next is "|_InvokeIP|ExpectedMarkerPosition|AcqId|" which contains expected marker positions.
5. The next row with "|_InvokeIP|InputSearchRegion|AcqId|" contains the pixel size information.
6. The "|_InvokeIP|MarkerSingleResult|AcqId|" row will yield the expected marker position and seed state in relation to tolerance diameter set before treatment.
7. Finally, convert from pixels to millimeters and calculate true geometric differences.

### 2.2 IMR‐calypso position comparison

While most users will only use a single method for intrafraction monitoring during treatment, Cetnar et al. performed a dual‐surrogate study to evaluate the accuracy of the IMR system.^1^ Electromagnetic monitoring technology such as Calypso® (Varian Medical Systems) can be used for real‐time motion monitoring during treatment.^7^, ^8^, ^9^, ^10^ This section is written for those interested in performing a similar comparison involving the transformation of coordinate systems for future work.

The Calypso system reports the fiducial positions in 3D in the linac vault coordinate system. The IMR system uses a kV x‐ray source and detector to image the patient and detects the Calypso beacon fiducials as markers on the 2D kV imager coordinate system. In order to be able to compare the positions reported by the two separate monitoring systems, we translated the positions of the fiducials from the 3D Calypso coordinate system to the 2D IMR coordinate system.

Three fiducials were analyzed for each patient during treatment. IMR 2D marker positions in the kV imager coordinate system, timestamp, and planar acquisition angle were recorded during treatment. Calypso System Data Converter Version 1.9 was used for 3D data extraction which reports 3D marker coordinates (X, Y, Z) of each fiducial. The synchronization of the two datasets was performed using the timestamps. If exact timestamp match was not found, the data set pairing was matched to the one with the shortest temporal difference. The average sampling time for our Calypso data reported coordinates was approximately 30 ms.

**FIGURE 4:**
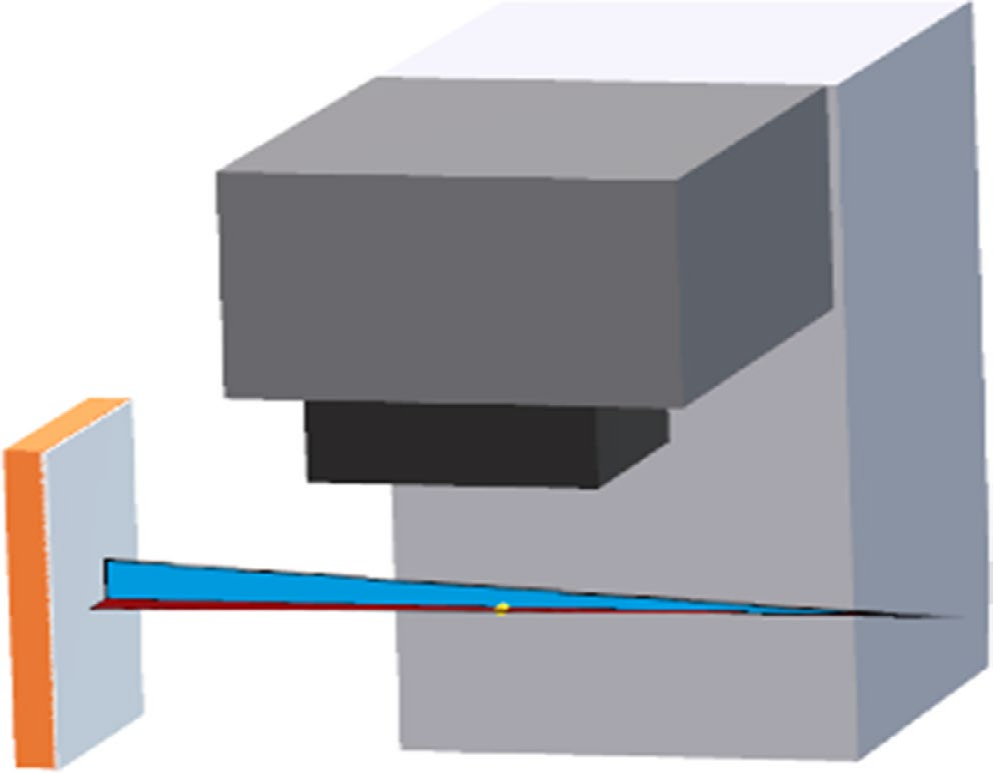
3D coordinates of the fiducials into the 2D planar kV imager coordinate system using a cone‐beam geometry at the linac

The gantry angle reported at the matched timestamp was used to project the 3D coordinates of the fiducials into the 2D planar kV imager coordinate system using a cone‐beam geometry shown in Figure 4.

The 90‐degree angle offset between the x‐ray source and the gantry angle was also taken into account when comparing data between the two systems. The projected 2D coordinates on the x‐ray detector (*u*,*v*) from Calypso are given by

$$u=\frac{d_{\mathrm{SDD}}*\left[‐x\;\mathrm{Sin}\left(\theta \right)+\;y\;\mathrm{Cos}\left(\theta \right)\right]}{d_{\mathrm{SAD}}‐x\;\mathrm{Cos}\left(\theta \right)‐\;y\;\mathrm{Sin}\left(\theta \right)},$$

$$v=\frac{d_{\mathrm{SDD}}*\;z}{d_{\mathrm{SAD}}‐\;x\;\mathrm{Cos}\left(\theta \right)‐\;y\;\mathrm{Sin}\left(\theta \right)}$$

where *d*_SDD_ and *d*_SAD_ are the *x*‐ray source‐to‐detector distance and the *x*‐ray source to rotation axis distance, respectively (*x*, *y*, *z*) are the 3D coordinates of the fiducials, and *θ* is the IMR planar image acquisition angle.

The labeling convention of the IMR system's markers and the Calypso system's fiducials are not necessarily the same. Therefore, an optimization routine to match the IMR markers to the Calypso beacon fiducials based on the calculation of the minimum distances was developed (in 2D detector coordinate system) for all pair combinations between the systems. The pair combinations with the minimum distance were interpreted as the matched IMR and Calypso marker and fiducial pairs.

**FIGURE 5:**
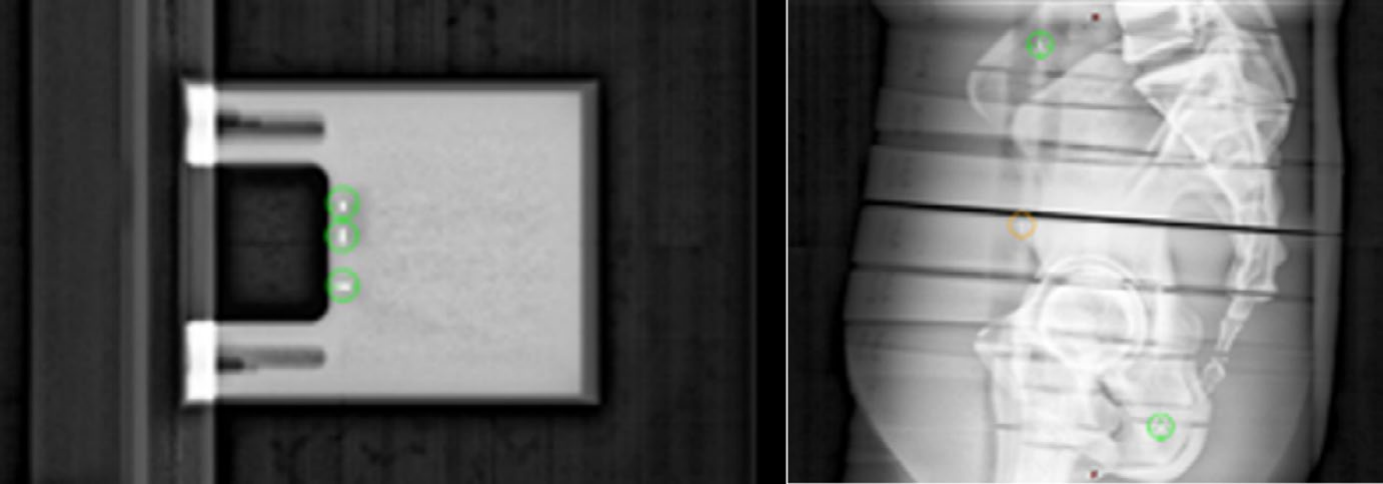
Phantoms used for algorithm validation included the Calypso Daily QA Phantom and an anthropomorphic phantom with Calypso beacons

The developed 3D to 2D projection code comparing IMR and Calypso data was validated with the data acquired using phantoms with embedded with Calypso fiducials shown in Figure 5.

## 3 RESULTS

**FIGURE 6:**
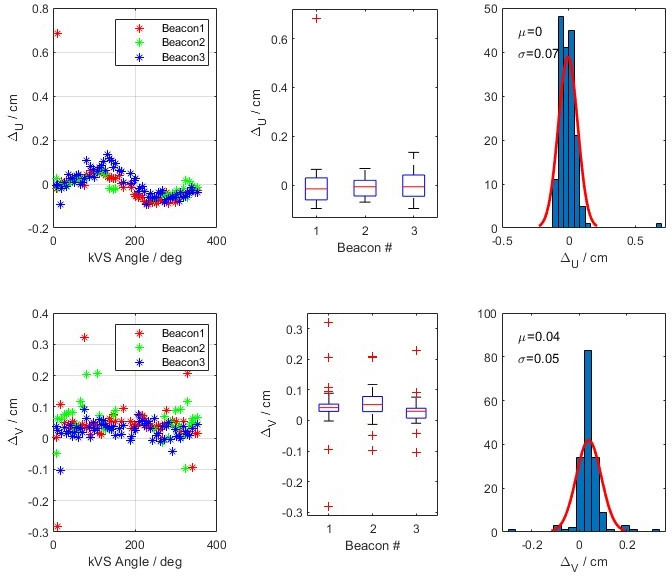
The calculated difference of u‐ and v‐coordinates of the fiducials from the Calypso and IMR data on the detector reference frame using an anthropomorphic phantom with embedded Calypso beacons

Validation of the 3D to 2D projection code for IMR and Calypso data compared calculated coordinates (*u*,*v*) of the fiducials with the Calypso data projected on the k*V* detector reference frame. The IMR detected coordinates and plotted in Figure 6 for the anthropomorphic phantom with embedded beacons. The top row shows the differences of the *u*‐coordinate between the Calypso and IMR coordinates with Δ*u* as function of k*V* source angle *θ*, the variation of this difference for each fiducial, and finally the variation of all fiducials in a single histogram. The bottom row is plotted for the *v*‐coordinate. The mean and standard deviation of the differences between the Calypso and IMR calculated *u*‐ and *v*‐coordinates are 0.0 (0.07) cm and 0.04 (0.05) cm, respectively. Validation testing was repeated using the Calypso Daily QA phantom yielding similar results calculated *u*‐ and *v*‐coordinates are 0.0 (0.04) cm and 0.04 (0.02) cm, respectively.

**FIGURE 7:**
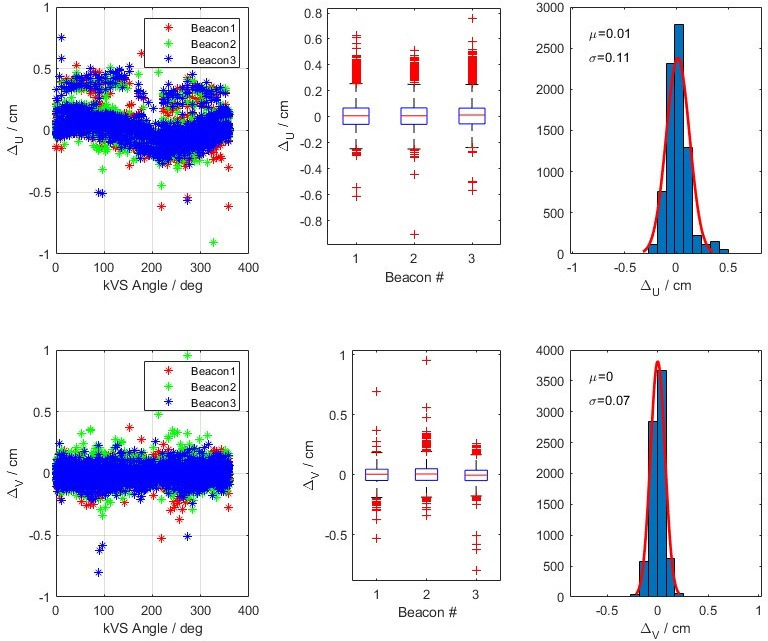
The developed 3D to 2D projection code comparing IMR and Calypso data was validated with the data acquired using patient data. This shows the combined data from 5 fractions, each with 2 arcs

Similarly, Figure 7 shows the differences of Δ*u* and Δ*v* as a function of kV source angle (θ), variation of the differences of each fiducial, and finally the variation of all fiducials in a single histogram in a patient cohort. The IMR algorithm was sometimes able to detect all three fiducials and marked them undetected or outside of the expected limit thresholds. In this analysis, we excluded the data when all three markers were not detected. For the patient data, the means of Δ*u* and Δ*v* are calculated as 0.01 cm and 0.00 cm, with standard deviations of 0.11 cm and 0.07 cm, respectively.

## 4 DISCUSSION

We faced several logistical challenges in the process of data extraction. The first involved the verification of timestamps between the two systems and establishing a method for synchronization for our analysis. The second is the awareness that updates to software can affect the output in the combined log files. It is recommended that a quality assurance program be developed for in‐house software after changes have been made.

Our final recommendation is to perform end‐to‐end tests using phantoms before using clinically to better understand how the data in the log files can be interpreted. We used vendor‐provided QA devices and anthropomorphic phantoms for our initial validation testing of our workflow which was helpful for making final improvements to our in‐house analysis. As an example, initial comparison data was not expected to have a large sinusoidal pattern between the two data sets. Upon investigation, we found differences in the offline review data for the imager position which we were able to correct and modify to better represent the data.

## 5 CONCLUSION

Through this process, we were able to develop ways to extract quantitative data helpful for us in understanding the accuracy of the IMR system and use it for process improvement within our clinical workflow. The current data available for clinical decision‐making is limited when using IMR. However, data is recorded in the combined log files, which can be utilized to better understand motion during patient treatment. Understanding data recorded during treatment within the combined log files can be helpful for retrospectively reviewing motion during treatment to continue to improve patient care.

## CONFLICT OF INTEREST

There are no conflicts of interest to disclose.

## AUTHOR CONTRIBUTION

Gavin Graeper – Made substantial contributions to the concept and design of the work, including acquisition, analysis, and interpretation of the data. Helped draft and revise the document through to its final submitted form. Agrees to be accountable for all aspects of the work. Ashley Cetnar – Made substantial contributions to the concept and design of the work, including acquisition, analysis, and interpretation of the data. Helped draft and revise the document through to its final submitted form. Agrees to be accountable for all aspects of the work. Ahmet Ayan – Made substantial contributions to the concept and design of the work, including acquisition, analysis, and interpretation of the data. Helped draft and revise the document through to its final submitted form. Agrees to be accountable for all aspects of the work. Michael Weldon – Made substantial contributions to the concept and design of the work, including acquisition, analysis, and interpretation of the data. Helped draft and revise the document through to its final submitted form. Agrees to be accountable for all aspects of the work.
